# Vocal divergence is concordant with genomic evidence for strong reproductive isolation in grasshopper mice (*Onychomys*)

**DOI:** 10.1002/ece3.5770

**Published:** 2019-11-06

**Authors:** Polly Campbell, Lena Arévalo, Heather Martin, Charles Chen, Shuzhen Sun, Ashlee H. Rowe, Michael S. Webster, Jeremy B. Searle, Bret Pasch

**Affiliations:** ^1^ Department of Integrative Biology Oklahoma State University Stillwater OK USA; ^2^ Department of Evolution, Ecology, and Organismal Biology University of California, Riverside Riverside CA USA; ^3^ Department of Developmental Pathology University of Bonn Bonn Germany; ^4^ Department of Biochemistry and Molecular Biology Oklahoma State University Stillwater OK USA; ^5^ Department of Forest and Conservation Sciences Forest Science Centre The University of British Columbia Vancouver BC Canada; ^6^ Department of Biology The University of Oklahoma Norman OK USA; ^7^ Macaulay Library Cornell Lab of Ornithology Cornell University Ithaca NY USA; ^8^ Department of Neurobiology and Behavior Cornell University Ithaca NY USA; ^9^ Department of Ecology and Evolutionary Biology Cornell University Ithaca NY USA; ^10^ Department of Biological Sciences Northern Arizona University Flagstaff AZ USA

**Keywords:** acoustic communication, behavioral isolation, contact zone, hybridization, reproductive character displacement, speciation

## Abstract

Behavioral barriers to gene flow often evolve faster than intrinsic incompatibilities and can eliminate the opportunity for hybridization between interfertile species. While acoustic signal divergence is a common driver of premating isolation in birds and insects, its contribution to speciation in mammals is less studied. Here we characterize the incidence of, and potential barriers to, hybridization among three closely related species of grasshopper mice (genus *Onychomys*). All three species use long‐distance acoustic signals to attract and localize mates; *Onychomys arenicola* and *Onychomys torridus* are acoustically similar and morphologically cryptic whereas *Onychomys leucogaster* is larger and acoustically distinct. We used genotyping‐by‐sequencing (GBS) to test for evidence of introgression in 227 mice from allopatric and sympatric localities in the western United States and northern Mexico. We conducted laboratory mating trials for all species pairs to assess reproductive compatibility, and recorded vocalizations from *O. arenicola* and *O. torridus* in sympatry and allopatry to test for evidence of acoustic character displacement. Hybridization was rare in nature and, contrary to prior evidence for *O. torridus*/*O. arenicola* hybrids, only involved *O. leucogaster* and *O. arenicola*. In contrast, laboratory crosses between *O. torridus* and *O. arenicola* produced litters whereas *O. leucogaster* and *O. arenicola* crosses did not. Call fundamental frequency in *O. torridus* and *O. arenicola* was indistinguishable in allopatry but significantly differentiated in sympatry, a pattern consistent with reproductive character displacement. These results suggest that assortative mating based on a long‐distance signal is an important isolating mechanism between *O. torridus* and *O. arenicola* and highlight the importance of behavioral barriers in determining the permeability of species boundaries.

## Graphical abstract

Divergence in behavioral traits is fundamental to the evolution of reproductive isolation and maintenance of species barriers. We characterized the incidence of, and potential barriers to, hybridization among three closely related species of grasshopper mice (genus *Onychomys*). We found little evidence for hybridization and a signature of reproductive character displacement in the mate attractions calls of the species pair with highest ecological and morphological overlap, highlighting the importance of behavioral barriers in determining the permeability of species boundaries.

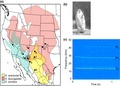

## 1 INTRODUCTION

Understanding the relative contributions of intrinsic (genetic, developmental) and extrinsic (ecological, behavioral) mechanisms to reproductive isolation is a long‐standing challenge in evolutionary biology (Coyne & Orr, 2004; Felsenstein, 1981; Mayr, 1963). Behavioral divergence is considered to be a major factor driving the evolution of reproductive barriers (Mayr, 1963; Turissini, McGirr, Patel, David, & Matute, 2017; West‐Eberhard, 1983). However, recent studies of speciation using next‐generation sequencing indicate substantial gene flow between closely related taxa despite strong intrinsic and/or extrinsic costs to hybridization (e.g., Cooper, Sedghifar, Nash, Comeault, & Matute, 2018; Rafati et al., 2018; Souissi, Bonhomme, Manchado, Bahri‐Sfar, & Gagnaire, 2018). Such findings emphasize the importance of understanding the behavioral mechanisms underlying assortative mating in determining the permeability of species boundaries upon secondary contact (Kopp et al., 2018).

Acoustic communication mediates social interactions in a wide variety of organisms. Divergence in acoustic signals used in mate recognition may contribute to premating reproductive isolation when costs of mismating are high (Mayr, 1963; Wilkins, Seddon, & Safran, 2013). Long‐distance signals may be especially sensitive to selection as receivers can detect and assess potential mates without incurring the costs of searching and/or direct physical encounters (Maynard Smith & Harper, 2004). Indeed, acoustic divergence predicts patterns of diversification in birds (Seddon, Merrill, & Tobias, 2008), and rapidly speciating insects often differ solely in acoustic traits (Henry, 1994; Mendelson & Shaw, 2005). In contrast, the contribution of acoustic divergence to reproductive barriers in mammals is considerably less studied.

**Figure 1:**
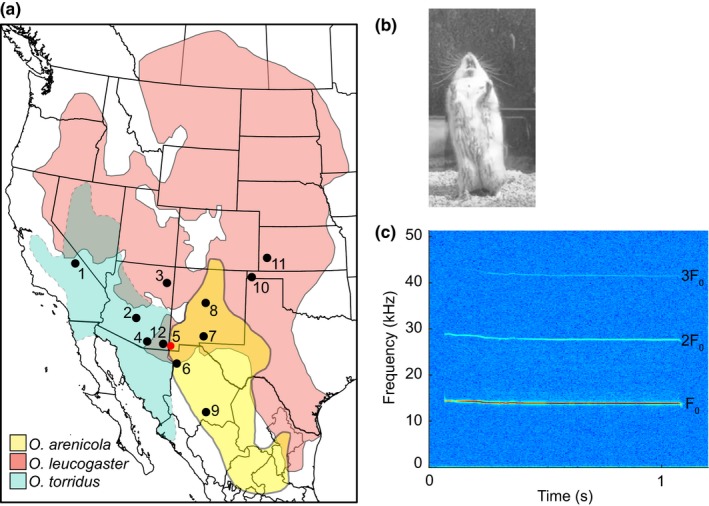
(a) The geographic distribution of grasshopper mice (*Onychomys*) showing areas of sympatry and the localities sampled in this study. Site 5 (indicated with a red dot) near Animas, NM is the contact zone between all three species and source of animals used in mating trials. Vocalizations were recorded from mice at sites 4 (*Onychomys torridus* allopatry), 5 (sympatry), and 7 (*Onychomys arenicola* allopatry). See Appendix S1 for sample sizes and additional locality information. *O. torridus* and *O. arenicola* range limits are indicated with dashed and solid lines, respectively; distribution map based on https://www.blueraster.com/smithsonian-mammals/. (b) Photograph of a northern grasshopper mouse (*Onychomys leucogaster*) vocalizing (D. Green). (c) Representative spectrogram of a long‐distance vocalization of *O. torridus*. F_0_ = fundamental frequency with harmonics (2F_0_, 3F_0_) at multiple integers of F_0_. The value of F_0_ explains the majority of variation among species and populations of grasshopper mice

Here, we test for evidence of gene flow between three closely related species of grasshopper mice (genus *Onychomys*), cricetid rodents in which long‐distance acoustic signals facilitate mate attraction and localization (Miller & Engstrom, 2012; Pasch, Tokuda, & Riede, 2017). Grasshopper mice inhabit prairies, deserts, and desert grasslands throughout the western United States, northern Mexico, and south‐central Canada (McCarty, 1975, 1978; Sullivan, Hafner, & Yates, 1986; Figure 1a). Members of the genus feed primarily on arthropods but also include small vertebrates and plant materials in their diet (Bailey & Sperry, 1929; Flake, 1973). As a consequence of their predatory lifestyle and large home ranges, both males and females produce loud advertisement vocalizations to announce their presence to potential mates and competitors over long distances (Frank, 1989; Ruffer, 1966). Animals often stand upright with open mouths oriented skyward to produce calls using airflow‐induced vocal fold vibrations (Pasch et al., 2017). The sexually monomorphic calls are innate (Pasch et al., 2016) and consist of a fundamental frequency (F_0_) and a series of harmonic overtones at integer multiples of F_0_ (Green, Scolman, Guthrie, & Pasch, 2019; Pasch et al., 2016, 2017; Figure 1b,c).

The largest of the three species, *Onychomys leucogaster*, is broadly distributed in the western United States, with northern and southern range limits reaching Saskatchewan, Canada, and Tamaulipas, Mexico, respectively (Figure 1a). The two smaller species, *Onychomys torridus* and *Onychomys arenicola*, are morphologically cryptic and were considered a single species until the pair was discriminated by fundamental number of the karyotype (Hinesley, 1979), allozymes (Sullivan et al., 1986), and mitochondrial haplotypes (Riddle & Honeycutt, 1990). Both *O. torridus* and *O. arenicola* co‐occur with *O. leucogaster* in arid regions throughout the western United States and northern Mexico but are largely allopatric with respect to each other (Figure 1a). However, all three species co‐occur in a narrow zone of contact in southwestern New Mexico.

Allozyme and karyotype data from the contact zone suggested that *O. arenicola* and *O. torridus* occasionally hybridize (Sullivan et al., 1986). Likewise, laboratory crosses between *O. arenicola* (identified as *O. torridus* prior to formal separation) and *O. leucogaster* from allopatric populations produced viable offspring, and backcross mice were morphologically indistinguishable from parental species (Pinter, 1971), raising the possibility that individuals with mixed ancestry have heretofore escaped detection.

In this study, we incorporated genomic, acoustic, and reproductive data to characterize the incidence of, and potential barriers to, hybridization in grasshopper mice. The genomic dataset combines field samples with museum tissues and includes the samples originally identified as hybrid animals (Sullivan et al., 1986). We used genotyping‐by‐sequencing (GBS) to obtain >88,000 SNPs and tested for evidence of historic and/or ongoing introgression in the New Mexico (NM) contact zone, together with additional allopatric and sympatric localities in the western United States and northern Mexico. A filtered set of SNPs was used to construct a nuclear phylogeny. We also sequenced an mtDNA fragment in selected samples from each locality to assess mitochondrial introgression. We recorded vocalizations from a subset of genotyped populations to determine whether population variation in call characters was consistent with estimates of introgression. Finally, we conducted mating experiments in the laboratory to determine reproductive compatibility among species.

## 2 METHODS

### 2.1 Genetic data and analyses

#### 2.1.1 Samples and DNA extraction

We obtained 260 tissue samples (83 *O. arenicola*, 88 *O. torridus*, 77 *O. leucogaster*, 12 not identified to species) from twelve localities (Figure 1b); 107 were field‐collected by authors of this study (B.P, A.R., P.C.), and 153 were museum tissue loans (Appendix S1). Tissues loaned from the Museum of Southwestern Biology (MSB; University of New Mexico, Albuquerque, NM) included samples that were only available as cryopreserved allozyme homogenates (see Appendix S1).

DNA was extracted from cryo‐ or ethanol‐preserved liver samples using either DNeasy Blood and Tissue or Gentra Puregene kits (Qiagen) according to the manufacturer's instructions. DNA from allozyme homogenate was extracted using a modified SDS extraction protocol. Briefly, 50 μl of the sample was added to 120 μl of ice‐cold homogenization buffer (0.1 M NaCl, 0.2 M sucrose, 0.01 M EDTA, 0.03 M Tris‐HCl, pH 8.0) and ground with a hand‐held pestle. The homogenate was mixed with 30 μl lysis buffer (0.25 M EDTA, 2.5% SDS, 0.5 M Tris‐HCl, pH 9.2) and incubated at 65°C for 30 min, followed by 1hr on ice with 20 μl 8 M potassium acetate. Remaining protein and cellular debris were pelleted in a cold microfuge (4°C, 15 min, 15,000 rpm [21,130 *g*]), and DNA was precipitated from supernatant with standard ethanol precipitation.

#### 2.1.2 mtDNA amplification and sequencing

Primers amplifying ~250 bp of *cytochrome c oxidase III* (*COX3*) in all three *Onychomys* species were designed from published sequences (Riddle, 1995) (Forward: 5′ GCTCTTTTATTAACATCAGGC 3′; Reverse: 5′ ATYCCTGTRGGTGGTCAGCA 3′). PCR reactions containing 10–20 ng DNA, 500 nM final concentration of each primer, and Platinum PCR SuperMix (Invitrogen) were run for 35 cycles with an annealing temperature of 53.5°C. Products were cleaned with the MinElute PCR purification kit (Qiagen) and sequenced in both directions on an Applied Biosystems 3730 DNA analyzer. Chromatograms were visualized with FinchTV (v 1.5.0, Geopsiza Inc.), and sequences were aligned and trimmed in Geneious Prime (Biomatters Ltd.). After finding evidence for contamination in a subset of museum samples from the NM contact zone based on *COX3* sequence (see Section 3), we sequenced the *COX3* fragment for all samples from that locality, together with 3–12 samples/species for all other localities.

#### 2.1.3 Genotyping‐by‐sequencing (GBS) and SNP calling

Library preparation and GBS sequencing of the 260 DNA samples was done at the Institute for Genomic Diversity at Cornell University, according to protocols established by the facility (Elshire et al., 2011). The enzyme PstI was used for digestion based on efficacy for GBS analyses in other rodents (e.g., Barbosa et al., 2018; White, Perkins, Heckel, & Searle, 2013). SNP determination was done with the TASSEL 5 GBSv2 pipeline (Glaubitz et al., 2014), and the reads were aligned to the *O. torridus* genome (GenBank, OnyTor_v1_BIUU) using BWA (Li & Durbin, 2009). Initially, a minimum of 10 sequencing reads was required for a SNP tag to be assigned. At the SNP discovery stage, the minimum locus coverage was set to 0.1 and the minor allele frequency was set to 0.01 in order to identify SNPs from the aligned tags. In total, 1,592,814,209 raw sequencing reads that passed quality control were analyzed, resulting in 551,770 SNPs before filtering. After filtering for minor allele frequency >5%, and missing data <10%, 88,494 SNPs (88K SNP dataset) remained for downstream analysis.

Thirty‐three allozyme homogenate samples from the NM contact zone were removed prior to further analysis due to either evidence for contamination based on mtDNA sequence (*n* = 13), insufficient/low quality reads (*n* = 10), or both (*n* = 10) (see Section 3).

#### 2.1.4 Haplotype network and phylogenetic analyses

A mitochondrial haplotype net was estimated in PopART (Leigh & Bryant, 2015) using the TCS algorithm (Clement, Posada, & Crandall, 2000). To build a nuclear phylogeny, we used a subset of 11,016 highly informative SNPs (11K SNP dataset) filtered by SNPSELECT software based on a pairwise linkage disequilibrium cutoff (Sun et al., 2019). We used maximum likelihood (ML) criteria in RAxML v8.1.18 (Stamatakis, 2014) with a GTR+I+G model of sequence evolution as determined in jModelTest 2.1.10 (Darriba, Taboada, Doallo, & Posada, 2012; Guindon & Gascuel, 2003). Node support was assessed using 1,000 rapid bootstrap replicates.

#### 2.1.5 Population structure analyses

To assess the degree of admixture between the three species we used *fastSTRUCTURE* (Raj, Stephens, & Pritchard, 2014), which applies a variational Bayesian inference approach to infer population structure from large SNP datasets. For this analysis, we used the full 88K SNP dataset. We determined the most likely range of model components (*K*) required to explain the structure within the dataset with a range from *K* = 1 to *K* = 10 as recommended (Raj et al., 2014), using the *chooseK.py* script provided by the authors. To ensure consistency of results, we replicated each run six times. The same approach was used to evaluate population structure within each species. Results were visualized using the R package ggplot2 (R Core Team, 2019; Wickham, 2016).

### 2.2 Acoustic data and analyses

#### 2.2.1 Data collection

We recorded calls of *O. arenicola* (*n* = 23; 13 females, 10 males) and *O. torridus* (*n* = 22; 11 females, 11 males) in allopatry (Organ Mountains, NM, and the Santa Rita Experimental Range, AZ*,* respectively) and sympatry near the contact zone in Animas, NM (*O. arenicola, n* = 21; 7 females, 14 males, and *O. torridus. n* = 19; 9 females, 10 males). The distance to sympatry was similar for both allopatric sites (221 km, Animas—Organ Mountains; 197 km, Animas—Santa Rita Experimental Range). Individually housed, wild‐captured animals in their home cage were placed within a semi‐anechoic sound cubicle for overnight (10 hr) recording in a mobile recording trailer (1976 13′ Scamp trailer) or in laboratory facilities at the University of Texas at Austin (allopatric animals) and Northern Arizona University (sympatric animals). We used 1/4″ microphones (Type 40BE, G.R.A.S.) connected to preamplifiers (Type 26 CB, G.R.A.S.) to record spontaneously produced vocalizations. Microphone response was flat within ±1.5 dB from 10 Hz to 50 kHz, and pre‐amplifier response was flat within ±0.2 dB from 2 Hz to 200 kHz. Microphones were connected to a National Instruments DAQ (USB 4431) sampling at 102.4 kHz to a laptop computer running MATLAB (version 2014a).

#### 2.2.2 Statistical analyses

Previous analyses of grasshopper mouse vocalizations indicate that calls are sexually monomorphic and F_0_ accounts for the majority of variation among species (Pasch et al., 2016, 2017). Thus, we extracted F_0_ in Avisoft SASLab Pro (version 4.2.27, Avisoft Bioacoustics, Germany; 256‐point fast Fourier transform [FFT]; Hann window with 50% overlap; frequency resolution 750 Hz, temporal resolution 0.67 ms). For each individual, we calculated averages from the total number of calls recorded ($\bar{x}$ = 30.2, range = 1–328). We used a linear mixed model with restricted likelihood estimation to test for the fixed effects of species (*O. arenicola* or *O. torridus*), degree of geographic isolation (allopatry vs. sympatry), and the interaction between species and degree of geographic isolation on F_0_, body mass, and residual F_0_ (obtained from a regression of log_10_ body mass on log_10_ F_0_). Individual identity was coded as a random effect. Conditional *F*‐tests using the Kenward‐Roger adjustment (Kenward & Roger, 2009) and post hoc Tukey HSD tests were used to assess differences among factors in JMP Pro (version 14.1.0, SAS Institute, Inc.). We also assessed if individual repeatability of F_0_ differed among species and populations by calculating intraclass correlation coefficients (ICC; Wolak, Fairbairn, & Paulsen, 2012) using the ICC R package (version 2.3.0, Wolak, 2013) in R version 3.6.1 (R Core Team, 2019). F_0_ was considered repeatable if the 95% confidence interval of ICC values excluded zero, and similar among species and populations if confidence intervals overlapped one another. Values are reported as mean ± *SD* in text.

### 2.3 Mating trials

We established a laboratory breeding colony to assess reproductive compatibility within and among species of *Onychomys*. Grasshopper mice captured in sympatry (Animas, NM) were transferred to the laboratory and housed in standard mouse cages (Ancare N40HT; 19″ × 10.5″ × 6 1/8″). Following at least 3 weeks of acclimation to the laboratory, we transferred a female and a subset of her nesting material into the home cage of a male shortly after the onset of nocturnal activity (2,100). We observed pairs for 2 hr to ensure compatibility defined as mutual oral and anogenital investigation and lack of agonistic barks and chases. A trained observer monitored animals daily to assess females for pregnancy and birth. Offspring were weaned at 30 days. We used ANOVA and post hoc Tukey‐Kramer tests to assess differences in litter size at weaning among conspecific and heterospecific crosses.

## 3 RESULTS

### 3.1 Hybrids are rare in nature

We recovered 27 unique haplotypes from mitochondrial *COX3* sequences. Haplotype diversity was 0.706 in *O. arenicola* (*n* = 40), 0.681 *O. torridus* (*n* = 47), and 0.888 in *O. leucogaster* (*n* = 31). Each species comprised a single cluster of haplotypes, with little evidence for geographic structure within species (Figure S1). There were no mismatches between mtDNA haplotypes and species identity based on nuclear genotypes.

**Figure 2:**
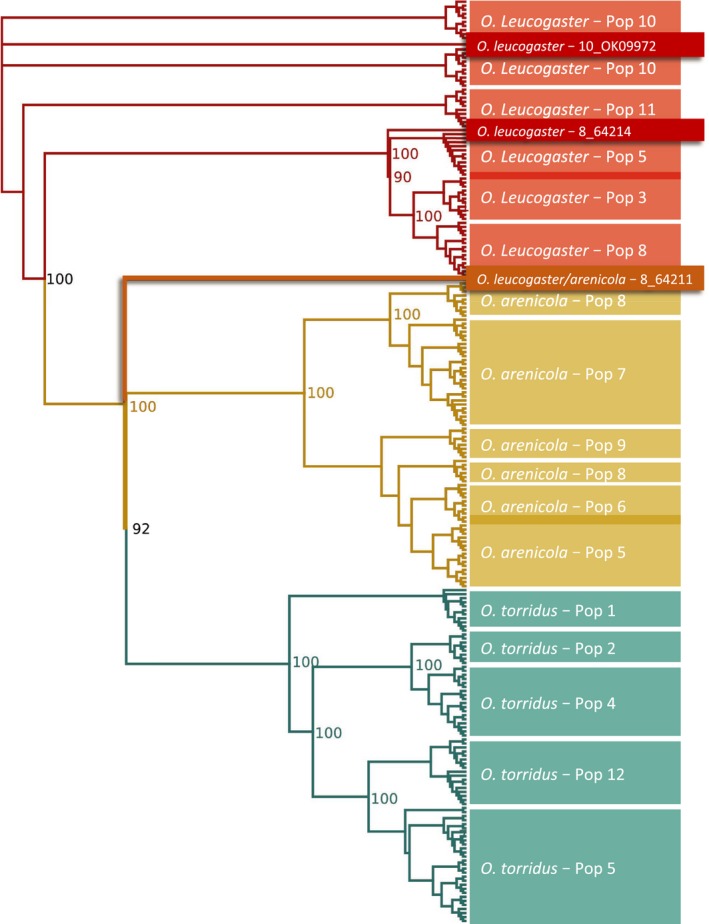
Phylogenetic relationships among grasshopper mouse (*Onychomys*) species and populations based on the 11K SNP dataset (see text), estimated using maximum likelihood criteria. Bootstrap support ≥90 is indicated for main clades. The tree is unrooted. Population (Pop) numbers match sampling sites in Figure 1a (see Appendix S1 for complete details). *Onychomys leucogaster* with evidence for historic introgression from *Onychomys arenicola* (10_OK09972), *O. leucogaster* admixed for eastern and western lineages (8_64214), and F_1_ hybrid between *O. leucogaster* and *O. arenicola* (8_64211) are highlighted. Darker shading between clades indicates that an *O. leucogaster* genotype sampled from Pop 3 was assigned to the Pop 5 clade and that an *O. arenicola* genotype sampled from Pop 5 was assigned to the Pop 6 clade

Final sample sizes for GBS‐based analyses were 227 (76 *O. arenicola*, 83 *O. torridus*, 67 *O. leucogaster*), of which 151 were from localities where two or more *Onychomys* species co‐occur. Phylogenetic analysis using the 11K SNP dataset recovered well‐supported (bootstrap = 100), species‐level clades, with *O. arenicola* sister to *O. torridus* and *O. leucogaster* basal to both in the unrooted ML tree (Figure 2). Within *O. arenicola* and *O. torridus*, the majority of genotypes from each locality formed monophyletic subclades, although not always with strong support (Figure 2). In *O. leucogaster*, genotypes from western Oklahoma and Kansas (localities 10 and 11, respectively, Figure 1a) were basal to all others, but relationships among locality‐specific subclades were poorly resolved. Notably, a single genotype (8_64211) from central New Mexico (locality 8, Figure 1a), identified in the field as *O. leucogaster* and carrying *O. leucogaster* mtDNA (haplotype u_8, Appendix S1)*,* was placed outside the *O. leucogaster* clade but basal to *O. arenicola* + *O. torridus* (highlighted in Figure 2).

**Figure 3:**
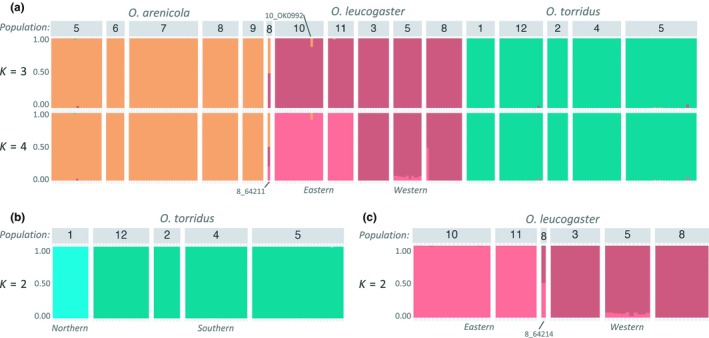
Genotypic clustering in grasshopper mice (*Onychomys*) using fastStructure (Raj et al., 2014). Colored bars represent individual ancestry proportions. (a) Population structure and species membership based on the 88K SNP dataset (see text) for *K* = 3 and *K* = 4. (b) Population structure within *Onychomys torridus* for *K* = 2. (c) Population structure within *Onychomys leucogaster* for *K* = 2. Population numbers match sampling sites in Figure 1a (see Appendix S1 for complete details). *Onychomys leucogaster* with evidence for historic introgression from *O. arenicola* (10_OK09972) and F1 hybrid between *O. leucogaster* and *O. arenicola* (8_64211) are indicated in (a); *O. leucogaster* admixed for eastern and western lineages (8_64214) is indicated in (c)

Bayesian clustering analysis in fastStructure identified three (*K* = 3) or four (*K* = 4) population clusters with *K* = 3 as the best model in half the runs and *K* = 4 as the best model in the other half (Figure 3a). In both cases, the three species formed distinct clusters, with *O. leucogaster* divided into two geographically structured lineages comprising samples from Oklahoma and Kansas (eastern) versus New Mexico and Arizona (western; Figure 3a) for *K* = 4. The intermediate genotype (8_64211) from central New Mexico highlighted in the ML tree was an F_1_ hybrid between *O. arenicola* and *O. leucogaster* that was assigned evenly (50/50) to the *O. arenicola* and *O. leucogaster* clusters in each of the replicate runs. This hybrid, and one pure *O. leucogaster* from the same locality (8_64214), was admixed for the eastern and western *O. leucogaster* lineages in the *K* = 4 model, suggesting secondary contact between these lineages in north‐central New Mexico (Figure 3). The only other sample with a strong signal of admixture was an *O. leucogaster* from western Oklahoma (locality 10, Figure 1a) with 10% assignment to the *O. arenicola* cluster (10_OK09972; Figure 3a). Since the collection locality is ~300 km beyond the northeastern distribution limits of *O. arenicola*, the introgression likely reflects historic gene flow. Within species, there was no signal of population structure in *O. arenicola* (*K* = 1), *O. torridus* genotypes from California (locality 1) clustered separately from Arizona and New Mexico genotypes (*K* = 2; Figure 3b), and *O. leucogaster* genotypes were split between Kansas/Oklahoma and Arizona/New Mexico (*K* = 2; Figure 3c) as in the *K* = 4 model with all three species.

We did not find evidence for recurrent or historic gene flow between any species pair in samples from the NM contact zone (locality 5, Figure 1a). Notably, visual examination of chromatograms from mtDNA sequence revealed “heterozygous” peaks at sites with species‐specific SNPs in 23/49 samples used for allozyme analysis by Sullivan et al. (1986), including two samples identified as putative hybrids between *O. arenicola* and *O. torridus* (Figure S2). This pattern was not found in any of the 34 tissue‐extracted samples from the contact zone, or in samples from any other locality. While apparent mtDNA heterozygosity can reflect heteroplasmy (e.g., Radojičić, Krizmanić, Kasapidis, & Zouros, 2015; Rokas, Ladoukakis, & Zouros, 2003) or nuclear gene copies (e.g., Antunes & Ramos, 2005; Liang, Wang, Li, Kimball, & Braun, 2018), cross‐contamination of heterospecific tissues during the original protein extraction for allozyme analysis is a more likely explanation.

### 3.2 Acoustic displacement in sympatry

**Figure 4:**
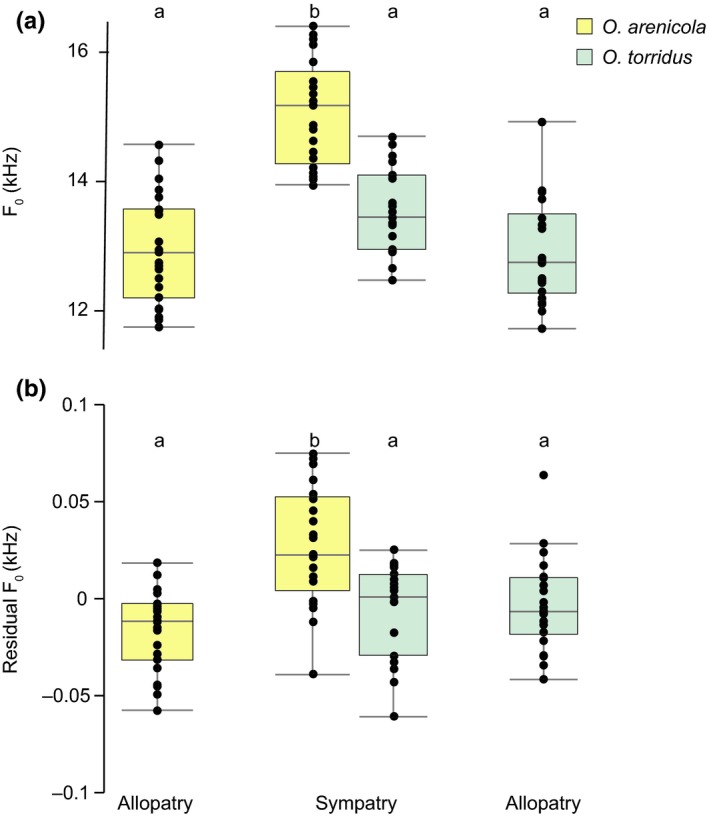
Population variation in vocalizations of grasshopper mice (*Onychomys*). (a) Boxplots and raw data depicting the fundamental frequency (F_0_) and (b) residual F_0_ (obtained from regression of log_10_ body mass on log_10_ F_0_) of *Onychomys arenicola* and *Onychomys torridus* calls in allopatry and sympatry. Groups with different letters above the boxplots are significantly different based on post hoc Tukey HSD tests

The F_0_ of vocalizations was highly repeatable within all individuals (ICC = 0.93, 95% CI, 0.91–0.95) and repeatability was similar between species and degree of geographic isolation (*O. arenicola* allopatry: 0.86, 95% CI, 0.79–0.93; *O. arenicola* sympatry: 0.86, 95% CI, 0.79–0.93; *O. torridus* allopatry: 0.89, 95% CI, 0.83–0.95; *O. torridus* sympatry: 0.74, 95% CI, 0.61–0.86). We found a significant species (*O. arenicola* or *O. torridus*) by degree of geographic isolation interaction on F_0_ (*F*_1,80.59_ = 19.62, *p* < .0001). Whereas F_0_ of allopatric *O. arenicola* (12.9 ± 0.8 kHz) and *O. torridus* (12.9 ± 0.8 kHz) was nearly identical, F_0_ was higher in sympatric *O. arenicola* (15.06 ± 0.8 kHz) compared to sympatric *O. torridus* (13.53 ± 0.7 kHz; Tukey HSD, *p* < .05; Figure 4a). This shift in voice was in part due to smaller body sizes of both species in sympatry (*O. arenicola*, 22.02 ± 5.7 g; *O. torridus*, 24.98 ± 4.3 g) compared to allopatry (*O. arenicola*, 28.03 ± 5.3 g; *O. torridus*, 33.26 ± 5.4 g; *F*_1,81.1_ = 38.17, *p* < .0001). However, even after accounting for mass (residual F_0_), calls of *O. arenicola* were significantly higher and thus more distinct from *O. torridus* in sympatry (*F*_1,80.89_ = 19.97, *p* < .0001, Tukey HSD, *p* < .05; Figure 4b).

### 3.3 Reduced reproductive output in heterospecific crosses

We found little evidence of behavioral incompatibility (aggression upon pairing) within conspecific pairs (6% or 2/32 in each of *O. arenicola*, *O. leucogaster*, and *O. torridus* trials) and only one incidence of incompatibility among all heterospecific pairings (*O. torridus* ♀ × *O. arenicola* ♂). The majority (73%–80%) of conspecific pairs successfully produced and weaned litters, whereas heterospecific pairs produced fewer or no litters (0%–25%) with slightly reduced litter sizes (ANOVA *F*_5,74_ = 4.55, *p* = .01 but *p* > .05 for all pairwise comparisons between conspecific and heterospecific crosses; Table 1). In addition, we found tentative evidence of intrinsic incompatibilities in a subset of hybrids in the form of stunted tails (Table 1). Tail abnormalities, a marker of developmental perturbation in mice (Neumann et al., 1994; Ruvinsky et al., 2002; Waddington & Carter, 1952), were never observed in conspecific offspring.

**Table 1:** Reproductive output from within‐ and between‐species crosses of grasshopper mice in the laboratory

Cross	Number of pairs	Litters weaned (born)^a^	Mean litter size at weaning ± *SD* (range)	% Success^b^
*Onychomys arenicola* × *Onychomys arenicola*	30	24 (27)	3.38 ± 1.0 (1–5)	80
*Onychomys leucogaster* × *Onychomys leucogaster*	30	23	4.22 ± 1.0 (2–6)	77
*Onychomys torridus* × *Onychomys torridus*	30	22 (26)	3.23 ± 0.6 (2–4)	73
*Onychomys arenicola* ♀ × *Onychomys leucogaster* ♂	20	0	0	0
*Onychomys leucogaster* ♀ × *Onychomys arenicola* ♂	20	0	0	0
*Onychomys arenicola* ♀ × *Onychomys torridus* ♂	20	5 (6)^c^	3.0 ± 0.7 (2–4)	25
*Onychomys torridus* ♀ × *Onychomys arenicola* ♂	20	4 (5)	4.0 ± 0.8 (3–5)	20
*Onychomys torridus* ♀ × *Onychomys leucogaster* ♂	20	2^c^	2.5 ± 0.7 (2–3)	10
*Onychomys leucogaster* ♀ × *Onychomys torridus* ♂	20	0		0

a Includes litters lost preweaning.

b Success indicates pairs that produced and weaned pups.

c Indicates that a pup from 1 litter was born with tail abnormalities.

## 4 DISCUSSION

Our findings suggest that hybridization is extremely rare in *Onychomys*. In our GBS analysis, we found neither evidence of introgression in the contact zone between all three species in southwestern New Mexico, nor evidence for introgression into or from *O. torridus* in any population. Thus, reproductive isolation between ecologically, morphologically and acoustically similar *O. arenicola* and *O. torridus* is apparently complete. Unexpectedly, we found evidence for occasional hybridization between *O. arenicola* and morphologically and acoustically distinct *O. leucogaster*: one well‐supported F_1_ hybrid between an *O. leucogaster* female and an *O. arenicola* male from a sympatric locality in central New Mexico, and one instance of historic introgression from *O. arenicola* into *O. leucogaster* from an allopatric locality in western Oklahoma. We discuss these findings in light of potential isolating mechanisms between species pairs.

With the availability of techniques such as GBS and RADseq that facilitate genome scans for introgression in nonmodel species, studies in contact zones have revealed varying levels of gene flow between closely related lineages across a broad range of taxa and genetic distances (e.g., Feder, Egan, & Nosil, 2012; Irwin et al., 2018; Shield et al., 2015; Souissi et al., 2018). Tests of introgression across 61 closely related animal species pairs, ranging from invertebrates to primates, identified a broad “gray zone” between 0.5% and 2% net synonymous divergence within which gene flow was likely to persist (Roux et al., 2016). Our divergence estimates for *Onychomys* species pairs fall within this range (Campbell and Arévalo, unpublished data), and we and others have shown that interspecific crosses can be achieved in the laboratory (Table 1; Pinter, 1971). So what explains the rarity of *Onychomys* hybrids in nature?

The simplest explanation is that signals used in mate recognition diverged in allopatry, such that behavioral isolation was complete upon secondary contact. However, call data for *O. arenicola* and *O. torridus* suggest an effect of sympatry on reproductive barriers. Whereas this species pair is morphologically similar and acoustically indistinguishable in allopatry, *O. arenicola* is smaller than *O. torridus* and produces higher frequency calls in sympatry. Ecological character displacement in body size to reduce resource competition is a common outcome among ecologically similar species in sympatry (Schluter, 2000; Pfennig & Pfennig, 2009), and shifts in voice may be a byproduct of change in body size (Boul, Funk, Darst, Cannatella, & Ryan, 2007; Titze, Riede, & Mau, 2016). Displacement in a mating signal can also evolve due to reproductive character displacement (RCD), selection to minimize reproductive interactions between species with similar signals (Pfennig & Pfennig, 2009). After controlling for allometry, the frequency of contact zone *O. arenicola* calls was higher than expected for their size, a pattern consistent with RCD to reduce heterospecific mate attraction.

Reinforcement of premating barriers due to selection against hybrids is implicated in many cases of RCD (Pfennig & Pfennig, 2009; Servedio & Noor, 2003). The occurrence of stunted tails in *O. arenicola*/*O. torridus* F_1_ hybrids produced in the laboratory, together with reduced reproductive output in interspecific relative to conspecific crosses, is suggestive of intrinsic incompatibilities that could reduce hybrid fitness in nature. However, support for reinforcement requires evidence of gene flow. Although we cannot rule out the possibility that *O. arenicola* and *O. torridus* occasionally hybridize in nature, we found no evidence for this in recently collected samples. In addition, our reanalysis of the samples of Sullivan et al. (1986) determined that the putative hybrids identified by these authors were artifacts of sample contamination. It is therefore unlikely that selection against hybrids contributes to premating barriers and putative RCD in call frequency between *O. arenicola* and *O. torridus*. Alternatively, the call frequency shift in sympatric *O. arenicola* may reflect response to selection to minimize reproductive interactions with *O. torridus*. Since both sexes call and calls are sexually monomorphic, divergence in call frequency and the frequency to which conspecifics respond could be explained by a simple matching rule whereby animals respond to call frequencies that match their own (Kopp et al., 2018). Under this model, selection to minimize reproductive interactions with *O. torridus* could drive call divergence if, for example, *O. arenicola* with lower frequency calls had lower reproductive success because they attracted (and were attracted to) *O. torridus* more often than animals with higher frequency calls. Even in the absence of mismating, response to heterospecific signals over long distances entail search costs that may similarly reduce fitness (Hammerstein & Parker, 1987).

Robust support for RCD as a driver of signal divergence in sympatry requires elimination of alternative processes (e.g., drift or another source of selection) that could produce the same pattern (Coyne & Orr, 2004). This is a significant challenge in any natural system and may be particularly difficult for *O. arenicola* and *O. torridus* because there is only one sympatric site and therefore no opportunity for replicate tests for displacement. Whereas replication of the pattern of vocal similarity between *O. arenicola* and *O. torridus* in multiple allopatric populations would provide additional support for RCD in sympatry, playback experiments with allopatric and sympatric mice will be critical to determining the effect of sympatry on response to heterospecific calls.

*Onychomys arenicola* and *O. leucogaster* are sufficiently ecologically dissimilar to coexist throughout the northern part of the range of *O. arenicola,* and there is no overlap in the F_0_ of the two species' calls (avg. F_0_ of sympatric *O. leucogaster* = 11.6 kHz, ~2 kHz lower than *O. torridus* and ~3.5 kHz lower than *O. arenicola*; Green et al., 2019). While laboratory crosses between animals from allopatric populations have produced viable hybrids (Pinter, 1971), none of our attempted crosses between wild caught *O. arenicola* and *O. leucogaster* from the New Mexico contact zone were successful. It was therefore surprising to find evidence for a low rate of hybridization (1/30 samples = 3.3%) at another sympatric locality in New Mexico (locality 8, Figure 1a). The hybrid was an F_1,_ and there was no evidence for interspecific admixture in the other genotypes from this locality, suggesting that hybrid‐mediated gene flow is rare or absent. However, the *O. arenicola* introgression in *O. leucogaster* from an allopatric locality in Oklahoma (site 10, Figure 1a) indicates that interspecific gene flow has occurred in the past. Resolution of the extent and history of hybridization between *O. arenicola* and *O. leucogaster* awaits further sampling in central and northeastern New Mexico.

*Onychomys* are unusual among rodents in producing long‐distance acoustic signals used in mate recognition within and, based on the pattern of acoustic RCD in *O. arenicola*, between species. However, it is likely that mediators of prezygotic isolation in other muroid rodents also operate in *Onychomys*. We consider three potential mechanisms. First, baculum morphology varies greatly among species (Burt, 1960; Schultz et al., 2016) and may cause mechanical isolation (Patterson & Thaeler, 1982). However, *O. torridus *from the contact zone in NM has the most distinct baculum (Sullivan et al., 1986) yet successfully mated with both congeners, whereas *O. arenicola* and *O. leucogaster* have similar bacula but never reproduced in our laboratory trials. Thus, we conditionally reject differentiation in baculum shape as sufficient for reproductive isolation in *Onychomys* (see Good, Demboski, Nagorsen, & Sullivan, 2003). Second, muroid rodents possess extraordinary olfactory abilities mediated by vomeronasal receptors (VRs) that bind ligands encoding information about species, sex, and status (Dulac & Torello, 2003). Although the functional diversity of VRs in *Onychomys* is unknown, sexually dimorphic midventral sebaceous glands that secrete pheromones (Pinter, 1985) likely play a key role in sexual and, potentially, species identification. Third, ultrasonic vocalizations (USVs) produced during male‐female interactions are important in coordinating reproduction in muroid rodents (Egnor & Seagraves, 2016). Unlike the sexually monomorphic long‐distance signals whose structure is constrained by detectability (Morton, 1986), the low‐amplitude USVs produced by *Onychomys* species in close‐distance mating contexts (Pasch et al., 2017; Riede, Borgard, & Pasch, 2017) likely contain redundant (species identity) and unique (sexual and individual) information (Holy & Guo, 2005; Musolf, Meindl, Larsen, Kalcounis‐Rueppell, & Penn, 2015). When coupled with olfactory cues, such signals may promote assortative mating in sympatric populations. Likewise, species differences in olfactory cues and close‐distance vocalizations may contribute to the reduced reproductive output in interspecific laboratory crosses (Table 1; Pinter, 1971).

Whereas speciation research has traditionally focused on single types of isolating mechanisms in a given system (e.g., genetic incompatibilities or ecological isolation; Coyne & Orr, 2004), recent work in invertebrates emphasizes the importance of multiple isolating mechanisms acting in concert (Dutta, Balakrishnan, & Tregenza, 2018; Gilman, Fowler‐Finn, & Hebets, 2018; Moran, Hunt, Mitchell, Ritchie, & Bailey, 2019). Here, we show that, contrary to prior suggestions (Sullivan et al., 1986), hybridization between *O. arenicola* and *O. torridus* either does not occur or is so rare that it is below the detection limits of our sample sizes. A pattern of RCD in long‐distance acoustic signals in sympatry suggests that selection to avoid the costs of attracting or being attracted to heterospecifics is strong in this species pair. In contrast, despite vocal dissimilarity, *O. arenicola* and *O. leucogaster* occasionally hybridize. Characterization of the full suite of signals used at different stages of the mate recognition process will provide a comprehensive understanding of the mechanistic basis of sexual isolation between *Onychomys* species pairs.

## CONFLICT OF INTEREST

None declared.

## AUTHOR CONTRIBUTIONS

Conceived and designed research, PC and BP. Performed research, BP, PC. HM, AHR, Analyzed data, BP, CC, LA, SS. Contributed facilities and reagents, MSW, JBS. Wrote paper, PC and BP with input from all authors.

## Data Availability

Mitochondrial DNA sequences have been deposited to Genbank (Accession Nos. MK995492–MK995518). Raw sequence data have been deposited to NCBI Short Read Archive (Accession No. PRJNA575734). Genotypic data have been deposited to Dryad (https://doi.org/10.5061/dryad.j2k683s).
