# Supplementary figures and images for: Vocal divergence is concordant with genomic evidence for strong reproductive isolation in grasshopper mice (Onychomys)
Source: Ecol Evol. 2019 Nov 6;9(22):12886–96. doi: 10.1002/ece3.5770 (PMC6875671; doi:10.1002/ece3.5770)

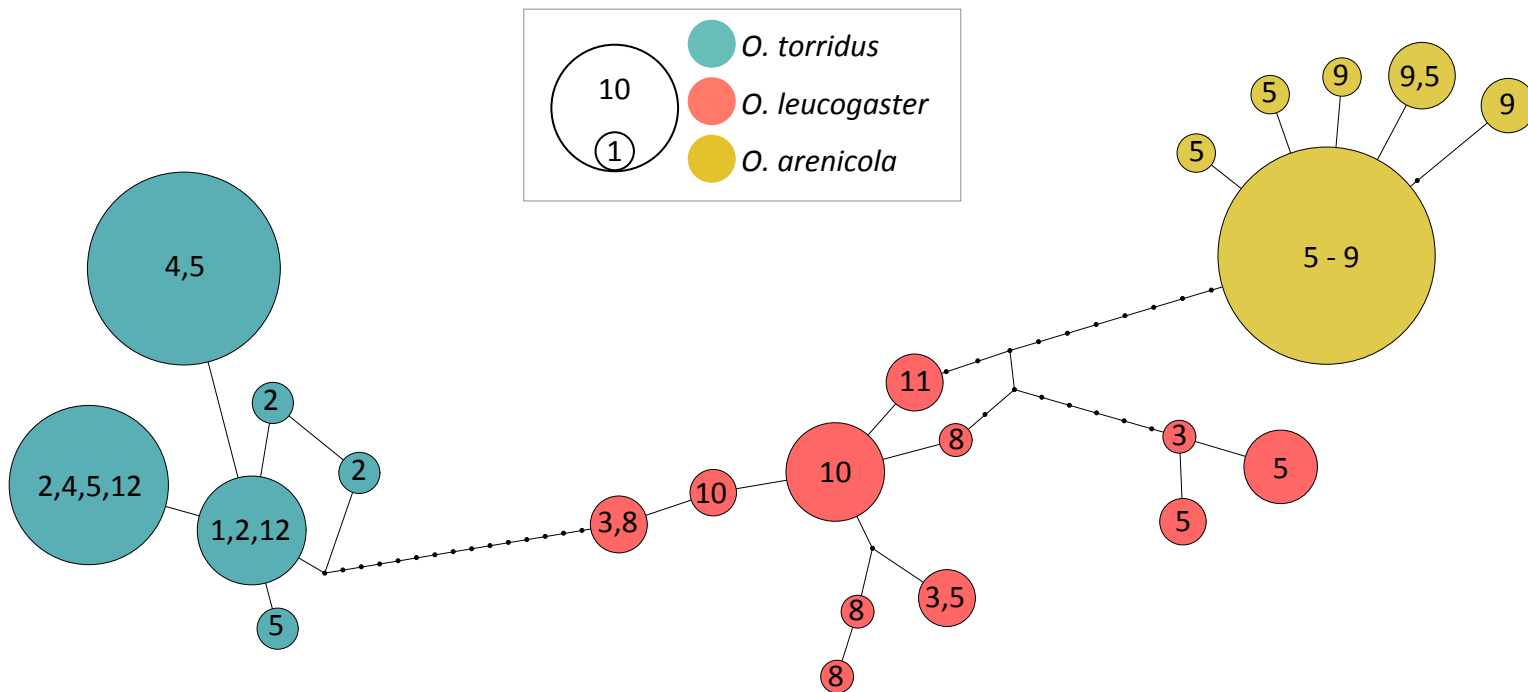

Supplement: Supplementary file 1 [file ECE3-9-12886-s001.pdf]

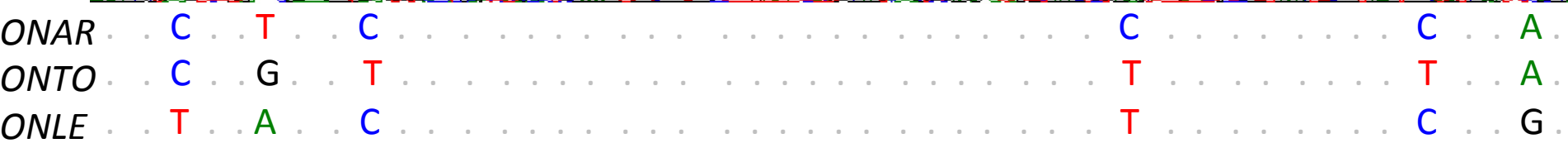

Supplement: Supplementary file 2 [file ECE3-9-12886-s002.pdf]
