# Supplementary material for: Vocal divergence is concordant with genomic evidence for strong reproductive isolation in grasshopper mice (Onychomys)
Source: Ecol Evol. 2019 Nov 6;9(22):12886–96. doi: 10.1002/ece3.5770 (PMC6875671; doi:10.1002/ece3.5770)
Supplement: Supplementary file 3 [file ECE3-9-12886-s003.docx]

**Supplementary Figure Legends**

**Figure S1.** TCS parsimony network for *COX3* haplotypes in grasshopper mice (*Onychomys*). Points on branches represent substitutions separating haplotypes; circle size is scaled by number of observed haplotypes. Numbers inside circles are sites where that haplotype was detected. 1, Lone Pine, CA; 2, Carefree, AZ; 3, Petrified Forest National Park, AZ; 4, Santa Rita Experimental Range, AZ; 5, Animas, NM; 6, Pancho Villa, Chihuahua; 7, Organ Mts., NM; 8, Sevilleta National Wildlife Reserve, NM; 9, Hildalgo del Parral, Chihuahua; 10, Rita Blanca Wildlife Management Area, OK; 11, Garden City, KS; 12, Chiricahua Mts., AZ.

**Figure S2.** Representative fragment of sequence from mtDNA gene encoding *COX3* for MSB 55059, identified by Sullivan et al. (1986) as a putative hybrid between *O. arenicola* (*ONAR*) and *O. torridus* (*ONTO*) based on allozyme data. “Heterozygous” peaks in the chromatogram are SNPs (colored bases) that are fixed between species; single peaks are conserved sites (gray dots). This pattern is best explained by cross-contamination of samples from all three species during the original protein extraction. *ONLE*, *O. leucogaster*.
